# Enabling research in care homes: an evaluation of a national network of research ready care homes

**DOI:** 10.1186/1471-2288-14-47

**Published:** 2014-04-05

**Authors:** Sue L Davies, Claire Goodman, Jill Manthorpe, Adam Smith, Natasha Carrick, Steve Iliffe

**Affiliations:** 1 Social Care Workforce Research Unit, King’s College London, London WC2R 2LS, UK; 2 Dementias and Neurodegenerative Diseases Research Network (DeNDRoN), 27 Old Gloucester Street, London WC1N 3AX, UK; 3 Department of Primary Care and Population Sciences, University College London, London NW3 2PF, UK; 4 Centre for Research in Primary and Community Care, University of Hertfordshire, Hatfield AL10 9AB, UK

**Keywords:** Dementia, Research networks, Long term care, Older people, Care homes

## Abstract

**Background:**

In the UK care homes are one of the main providers of long term care for older people with dementia. Despite the recent increase in care home research, residents with dementia are often excluded from studies. Care home research networks have been recommended by the Ministerial Advisory Group on Dementia Research (MAGDR) as a way of increasing research opportunities for residents with dementia. This paper reports on an evaluation of the feasibility and early impact of an initiative to increase care home participation in research.

**Methods:**

A two phase, mixed methods approach was used; phase 1 established a baseline of current and recent studies including the National Institute for Health Research portfolio. To explore the experiences of recruiting care homes and research participation, interviews were conducted with researchers working for the Dementia and Neurodegenerative Diseases Research Network (DeNDRoN) and care home managers. In phase 2, four DeNDRoN area offices recruited care homes to a care home network for their region. The care home networks were separate from the DeNDRoN research network. Diaries were used to document and cost recruitment; DeNDRoN staff were interviewed to understand the barriers, facilitators and impact of the care home networks.

**Results:**

Thirty three current or recent studies were identified as involving care homes as care home specific studies or those which included residents. Further details of care home recruitment were obtained on 20 studies by contacting study teams. Care home managers were keen to be involved in research that provided staff support, benefits for residents and with minimal disruption. In phase 2, 141 care homes were recruited to the care home research networks, through corporate engagement and individual invitation. Pre-existing relationships with care homes facilitated recruitment. Sites with minimal experience of working with care homes identified the need for care home training for researchers.

**Conclusions:**

Phase 1 review revealed a small but increasing number of studies involving care homes. Phase 2 demonstrated the feasibility of care home research networks, their potential to increase recruitment to research and develop partnerships between health services and care homes, but highlighted the need for care home training for researchers.

## Background

In England commercial companies and not for profit organisations are the main formal providers of long term care (in care homes, with and without on-site nursing) for older people. Approximately 17% of people aged over 85 live in a care home and the number of residents is projected to rise [1]. In March 2012 there were 13,134 residential care homes with 247,824 beds in England, and 4,672 nursing homes with 215,463 beds [2]. The majority of residents are female, in their mid-eighties, with multiple morbidities, and have a median life expectancy of 2–3 years in residential care and 1–2 years in nursing homes [3-5]. This is a heterogeneous population with a wide range of health care needs. However, it is estimated that dementia affects at least 75% of residents and is severe for over 30% of those residents who have it [6,7].

There is a long if scattered history of health care professionals working with care homes (and specifically nursing homes) to improve the quality of staff education and to enhance evidence based care. Initiatives have included the NHS Institute for Innovation and Improvements Care Home Programme [8] teaching nursing homes [9,10] and academic care homes [11], but the focus has not been specifically on research in long term care facilities. None of these initiatives have been implemented nationally, and it is only in the last five years that research in care homes has markedly increased [12]. Compared with ageing research overall, research in this sector remains relatively underdeveloped [13,14]. This may be explained by the difficulties in recruiting older people in care homes to studies, and it is increasingly recognised now that there is a need to tailor research approaches to this setting. Such tailoring needs to acknowledge the care home as a person’s home, the importance of the care home’s organisational culture, and the resources that are required to maximise participation [15-19].

Since 2006 a national infrastructure of both general and topic specific (e.g. for dementia and neurodegenerative diseases, cancer, stroke, diabetes) clinical research networks, have worked to increase patient recruitment to research; to integrate research with NHS provision; to support research activity and to build research capacity [20]. Within England, the networks are organised by region to provide geographic coverage and exploit pre-existing networks of clinicians and researchers. In recognition of care homes as the main providers of long term care for people with dementia, the Ministerial Advisory Group for on Dementia research [21] recommended that a research network of care homes should be created. The purpose of the network was to improve the consistency of support for research outside the NHS, and ensure that residents with dementia, a group often excluded from research or seen as difficult to recruit, had more opportunities to participate in relevant studies. In 2012 the Dementia and Neurodegenerative Diseases research network (DeNDRoN) set up an online resource for researchers working in care homes, care home staff, residents and family members and began to build a network of “research- ready“ care homes. The overall project title chosen was ENRICH – Enabling Research in Care Homes.

**Figure 1:**
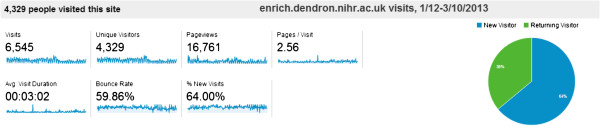
Summary of ENRICH toolkit uptake in 2012.

A working group was established to develop the online resource and oversee the development of guidelines for the recruitment of the care home network. Membership spanned a range of care home organisations, charities, carers and resident representatives, clinicians, researchers; the working group was supported by a project manager. Over eight months resource materials and illustrative case studies of recent studies for the online website were developed and circulated for consultation and review. Topics addressed common research challenges encountered in care homes, (for example obtaining consent, methodological approaches; a tariff to guide reimbursement for care staff time; questions that care home managers, residents and relatives should ask when approached to participate in a research study, and different strategies to involve people with dementia in research). The online materials were piloted, and the web site was launched in February 2012, and a dedicated section on the ENRICH care home networks, including online membership was added in August 2012 [22]. Google statistics were used to monitor the website (see Figure 1).

This paper describes the development of a national network of research-ready care homes to increase research activity and capacity in England, and presents the findings from an evaluation of the pilot study in its first year (January to December 2012). The evaluation was reviewed and approved by the Dyfed Powys Research Ethics Committee reference (12/WA/0134) and social care governance was obtained from the relevant local authorities.

## Method

A two phase multi-method approach was used to set up and evaluate the first year of the ENRICH network. Phase 1 sought to establish a baseline measure of current research activity in care homes in England and to explore care homes’ priorities for research. Phase 2 aimed to establish the minimum requirements for an effective research enabled care home, and to evaluate the initial impact of the ENRICH network on study recruitment, care home staff engagement and costs. Written informed consent for participation in the study was obtained from all participants.

### Phase 1 – Research in care homes overview

Studies that were care home specific or had recruited from care homes in addition to other settings, were identified to establish a baseline of care home research activity prior to setting up the care home network. As the focus was on current research, the search was limited to the previous year, which provided the opportunity for the researchers to be contacted for further study related information. Three main data sources were used for this review;

1. Online searches of the NIHR and Social Care Research Register portfolios of research studies

2. Interviews with local DeNDRoN research network staff

3. Interviews with care home managers or senior staff of homes that had already participated or expressed an interest in research, and focus groups with their corresponding relatives’ and residents’ groups.

This was supplemented by emails requesting information on current research to the national care home research and development forum, professional organisations (e.g. the British Geriatrics Society, Royal College of Nursing), National Care Home Research and Development Forum, post graduate groups and funders of research with older people. Where possible, the Principal Investigators from the studies identified were contacted by email or phone, to establish how long it had taken to recruit care homes, how many had been approached, and what, in their views, had facilitated and inhibited care home involvement.

Semi structured interviews conducted with DeNDRoN staff and care home managers were used to generate information on care home involvement in research and the barriers and facilitators for a care home to be effectively ‘research enabled’. Research managers from each of the four DeNDRoN pilot sites were interviewed prior to the setting up of the care home networks, to elicit the number of current and recent studies involving care homes, how many care homes they had regular contact with and the nature of their relationships with them. Care home managers were identified for interviews in two ways; either those who had expressed an interest in ENRICH following press releases, or, as their care homes were recruited to the care home networks, they were invited to take part in an interview. These interviews focused on their experiences of being involved in research and their expectations of a possible research ready care home network. Two focus groups, one with residents and another with relatives, to be recruited through care homes that agreed to join the care home networks, were planned to obtain their perspectives and experiences of research in care homes.

### Phase 2 – Network development

Phase 2 of the study focused on a process evaluation of the setting up of the ENRICH care home research network, by DeNDRoN staff in four pilot sites in different areas of England, in addition to their existing work. These sites were self-selected from a possible eight local research networks; four agreed to participate in the pilot. The aim of the pilot was to recruit up to 40 care homes across the four sites within the first six months of its operation (May to October 2012).

Guidance for recruitment of care homes to the ENRICH network was developed by the project working group and regular teleconferences were held to give support from the project manager. Care homes could choose from one of three possible levels of involvement in the network, ‘Support’, ‘Assist’ and ‘Deliver’, to allow for differences in their capacity to engage in the research process. These levels are defined as follows:

1. ‘Support’ - care homes agree to promote any studies to residents and staff

2. ‘Assist’ – care homes will actively identify residents and staff who might be eligible to join studies

3. ‘Deliver’ – care homes will participate fully to assist and support the delivery of research studies

In order to cost the setup of the network, DeNDRoN staff at each pilot site kept a structured diary record of the care home network recruitment process which included details of how care homes were identified, type and number of contacts and staff time spent on the project. At the end of the six month recruitment period, semi -structured interviews were conducted with DeNDRoN staff to explore their experience of setting up the care home network pilot, including the obstacles and facilitators to the process.

### Analysis

Data from phase 1 was used to describe the current arrangements that facilitate research in care homes as well as the number and range of current and recent studies involving care homes, their focus and methods. In phase 2, the different data sources, diaries and interviews, were synthesised to provide a descriptive account of the process of setting up the ENRICH care home network, the related resources and costs, numbers of care homes recruited, facilitators and barriers to care home recruitment, and perceived impact on care homes.

## Results

### Phase 1

#### *Baseline of care home studies*

Thirty three studies involving care homes were identified through online searches, interviews with DeNDRoN research network staff and care home managers, and email requests to researchers. These studies were either current or had been completed within the last 12 months. In total, 18 studies were identified through the NIHR portfolio, six through the Social Care Research Register and the remaining nine were located in response to the email alerts, voluntary sector groups supporting older people’s research, through personal networks and contacts of the ENRICH project board. Overall, 23 of the 33 studies were wholly based in care homes, 13 (39%) were studies of an intervention, including 9 RCTs; 14 (42%) were observational studies, and 9 (27%) involved training for care home staff. It was not possible to classify six studies as there was not enough information available. Additional file 1: Table S1 provides a breakdown of the focus, methodology and source of the studies confirmed to have care home involvement, and where available the number of care homes and residents that were recruited.

Twenty researchers (61%) from the studies identified through the searches responded to requests by email or telephone interview for more information about the experience of recruiting care homes to research studies. The number of care homes recruited to studies ranged from 3 to 63, with the number of residents recruited ranging from 10 to 572. Researchers reported a wide variation in the number of care homes that agreed to participate compared with the total number that were approached, this ranged from 27% to 73% across the individual studies. For example, members of the research team working on one large scale study visited 120 care homes in order to recruit 48, the recruitment took between five and six months. A smaller study contacted 44 care homes of which 32 declined before the researchers could successfully recruit six care homes. Overall researchers had to approach at least 40 per cent more care homes than they needed in order to recruit the required number. Most care homes were located via the Care Quality Commission (CQC) (regulator) website and recruited through ‘cold calling’, although a small number of researchers had established relationships with care homes. There were recurring themes about the length of time to recruit care homes, and linked issues about governance arrangements. Table 1 gives a summary of some of the researcher’s views on the barriers and facilitators to recruitment and on-going care home involvement in research.

**Table 1:** Barriers and facilitators to recruitment identified by researchers

**Barriers**	**Facilitators**
Studies that require a large time commitment form care home staff	Producing a brochure outlining the study and its potential benefits
Staff turnover – some care homes that agreed to take part in studies dropped out after a change of management	Involving relatives in the research process as much as possible
Care home organisations not giving permission for studies to be conducted in their care homes	Using a designated team to work with the care home residents and staff, drawing on clinicians that were linked to the care homes
Research protocols that do not fit with the care home’s mode of operation	Building in some research training for care home staff into the study process

*Prior* relationships with care homes increased the likelihood of their participation, especially where the immediate benefits of research for both staff and residents were apparent for example, training for care home staff or therapy for residents.

#### *Interviews with DeNDRoN research managers prior to the care home network*

Initial interviews were conducted with managers or their equivalent, from each of the four DeNDRoN local offices prior to them setting up their respective research enabled care home networks. (Further details are given on how the care home networks were set up in the phase 2 findings). Across the four LRNs, a total of 10 studies were identified that were either currently recruiting from care homes or had done so recently, this ranged from 2–4 studies for the individual DeNDRoN offices. These studies were included in the total baseline number.

These four DeNDRoN offices had established different relationships with the care homes in their locality depending on their degree of contact through these previous research studies. Three had been involved in recruiting care homes to large scale studies, and consequently had set up a database of care homes including a sub-group of those with a particular interest in research. However, only one office had actively maintained regular contact with a small group of care home via a research forum and sending out newsletters and details of research and development training days.

#### *Interviews with care home managers*

Interviews were completed with nine care home managers from the 15 care homes that were recruited through the ENRICH pilot sites, and one care home manager who contacted the ENRICH office directly. Five of the managers had some experience of research themselves. Managers consistently identified the perceived benefits of participation as an opportunity to improve the quality of care through research, raise staff awareness of new developments, establish links with other care homes in their locality, improve access to training and providing the opportunity to identify questions and areas for research.

*‘I think you have access to different parts of the country and you can share ideas. Yes we’re a company and there are five homes but that’s quite isolating as well. So if you network you can look at how things work in other homes and therefore develop the service you are providing…. Its additional support so you’re not isolated’* ***. Care home manager 5 talking about the idea of a care home network***

One care home manager commented that becoming a member of ENRICH had been viewed favourably by the regulator as an indicator that the care home was committed to evidence- based practice and transparency in its work. Overall, membership of the network was perceived as both a way of accessing research and also networking with other care homes for mutual support. However, there was some lack of clarity about the role of the pilot sites and how ENRICH would introduce studies to the care homes. Managers highlighted four main areas of support they would need in order to engage in research. These were support with recruitment when explaining studies to residents, early involvement of residents’ families, data collection that took account of residents’ needs, and tailored information and support for care home staff. A further facilitator was the identification and involvement of specific research staff within the care home to support data, collection.

‘I think keeping it fairly informal as it can sound scary to someone who’s never been involved in research before - you could have a group of residents, families who are often older people and they might think ‘that’s too much for me I couldn’t possibly do that’. So it’s about keeping it tailored to the right level because if you’ve just got a group of qualified nurses then you would pitch it slightly differently’. Care Home Manager 1

Care home managers reported that they were keen to be involved in research but wanted their participation to fit with their workload and to retain a level of control over their involvement in the research process. Barriers to care home recruitment included lack of time and interest in research, and study interventions that did not fit with the care home’s practice or routine, for example, drug trials that necessitated a change in the medication regime. Care homes were unlikely to have the capacity to take part in more than one study at any one time. The corporate nature of much of the care home sector could mean that managers might be willing to take part but were unable to without the permission of senior management. Changes of management and staff turnover in general were also common reasons for care home managers withdrawing from studies. Both care home staff and researchers reported that the recruitment process was facilitated by the use of clear explanations of studies through written material and verbal presentations. Another priority was the early involvement of residents’ relatives in the study, especially when residents lacked decision making abilities and consultees needed to be involved in the consent process.

Care homes are independent providers and it was therefore surprising that there were no examples of researchers offering, or home managers expecting, financial compensation for care home involvement in research, despite the fact that some interventions involved significant levels of input from care home staff. For example, studies could run for over a year, including follow up data collection, and could involve up to three or more care home staff in each care home in the research activity. Other examples of care home staff involvement in studies included, identification of eligible residents, distribution of study letters to residents and their relatives, explaining the study to residents, liaising with residents and the study team, facilitating access to residents and their care home notes, collection of data with and without the researcher, and participating themselves in individual and group interviews. All of which was assimilated into the working routine of the care homes with no reimbursement for staff time.

Phase 1 revealed a range of research activity in care homes, the need to spend time on recruitment and the expertise that some research teams had built up on working with care homes, mainly by trial and error. Those interviewed supported the potential of a research ready care home network if it could streamline the recruitment process and maintain relationships with care homes between studies. It also highlighted the goodwill of care homes to support research. It was not possible to carry out focus groups with residents and their families within the allotted time frame.

### Phase 2 – The care home network findings

In phase 2, the process of establishing and recruiting to the care home networks was documented and monitored (March to October 2012). After eight months 141 care homes had been recruited to the ENRICH network, 15 were recruited directly through three of the four pilot sites and 125 care homes through one national corporate provider. One care home joined the network online through visiting the ENRICH website. One pilot site dropped out due to lack of available staff to work on ENRICH.

The pilot sites used five recruitment mechanisms with varying success including, building on prior working relationships with care homes, cold calling with follow up, holding group meetings for care homes and online membership. Corporate membership was negotiated centrally by DeNDRoN based on liaison with one corporate provider. It added another level of administration to the recruitment process, as it was agreed that all potential studies would be initially screened by the corporation to ensure a fit with company protocols and priorities. Once corporate endorsement was obtained, suitable care homes would be identified at which point, care home managers could choose to participate or not, albeit with the full support of their organisation.

The care homes recruited to the network ranged in size from 11 to 60 beds, ownership reflected a range of providers from large for profit and medium care home organisations as well as owners of only one home. All care homes were registered as able to provide dementia care and most had achieved high ratings on their CQC inspections. The total number of residents was 514. Only one regional pilot area achieved the target recruitment of 10 care homes and did this in three months. This site had a pre-existing working relationship with care homes and had maintained contact them through phone calls, newsletters and invitations to training and events. Cold calling to care homes that had no existing relationships with the pilot sites, with follow up, group discussions and visits were labour and resource intensive for example, staff in one pilot site made 55 phone calls in order to recruit four care homes. Visits could take up to half a day including travelling time and mirrored the experiences described by the researchers in phase 1. However, once a care home had expressed interest in membership, they all agreed to join the network.

Two sites were still recruiting at the end of the period of data collection. Across the three sites between three and seven people were involved in recruiting care homes and included researchers, administrators and clinicians. Interviews with participant DeNDRoN managers highlighted the difficulties of incorporating care home recruitment into existing workloads. In the two sites that had minimal experience of working with care homes, there was a perceived need for staff training and support to understand care home culture and operation. Based on records of staff time, estimated travel costs and related expenses (e.g. group meetings and catering) the average cost of recruiting one care home to the network in the pilot site with pre-existing relationships was £253.33; the cost of recruiting a care home across the other sites could not be calculated as they were still recruiting at the end of the evaluation.

## Discussion

We identified 33 current or recent studies, through the NIHR and Social Care Research Register portfolios and other sources, which were care home specific or recruited residents from care homes. This review generated a baseline that can be used as a measure of care home research activity. The number of studies was higher than we anticipated, which may reinforce the findings about the recent increase in RCTs in care homes [12]. It also highlighted the scope for expansion of care home research activity and supported the need for the development of a care home research network to streamline the identification and recruitment of care homes.

Out of the 10 care home managers that were interviewed in phase 1, only half of them had participated in research previously, but overall the experience had been positive. Contrary to expectations, financial incentives were not a prerequisite for participation, but support and sensitivity to the needs of residents with dementia were. The potential for mutual benefit was a recurring theme in both phases of the evaluation,. Other perceived and reported benefits included the impact on staff education, improved care, positive feedback from CQC inspectors, increasing dementia research, and the opportunity for staff to voice their opinions, concerns and ideas for improving residents’ care.

Managers expressed a need to be able to control the level of participation and engagement in research. This finding reinforced the value and flexibility of the three different levels of participation on which they are currently enrolled into the care home research network. Time constraints were evident, even when the benefits of being involved in research were perceived by care home staff as far outweighing the negative aspects. It is a recurrent theme in care home research and other literature that they are often isolated from the communities in which they are based and the wider systems of health care [23]. An unanticipated impact of ENRICH was its linkage role with other care homes providing a focus that did not pose a competitive threat.

Even with project management and the involvement of support staff, the establishment of the care home network took longer than anticipated. It took eight months to recruit 141 care homes to the ENRICH care home network. A number of recruitment strategies were used to recruit care homes to the networks, including a corporate membership process which was developed centrally by DeNDRoN through liaison with one corporate care home provider. Corporate level engagement provided a quick route for making contact with care homes but introduced a further bureaucratic layer of permissions that did not necessarily guarantee care home manager engagement and support. A full costing of the pilot set up process was not possible but based on the completed site the average cost of recruiting one care home appears to have been relatively inexpensive. The set up process demonstrated a need for multiple approaches that reflect the heterogeneity of the care home sector. Most care homes in England have less than 10 residents and do not have on-site nursing, although this situation is rapidly changing as economies of scale mean care homes are increasing in size and joining larger chains [1].

To our knowledge the ENRICH network is the first national network that is supported by an online resource for researchers and participants and is integrated with a national infrastructure of clinical research networks. It was initiated as part of a national strategy to improve the reach and quality of dementia research [21]. Internationally, examples of other initiatives in the United States, the Netherlands, Norway and Australia have focused on collaborations and schemes to support teaching and innovation in care with nursing homes [24]. These are however, localised and care homes can only join through the organising institution.

Following the completion of data collection, another five regions have established local Research-Ready care home networks, and have begun to recruit homes; the total number of care homes involved currently is 509 care homes recruited within the first 18 months of operation. This includes 86 individually recruited care homes, a further corporate provider with 73 care homes, and a care home network has also been set up in Scotland. Since its establishment, the ENRICH care home network has supported approximately 18 new studies to undertake research in care homes. In addition to resident recruitment this has also addressed care home staff engagement through their inclusion in study writing groups, trial steering committees and the review of study protocols. The network is expected to double in size within the next 12 months, and will support recruitment to new studies funded by the Economic and Social Research Council (ESRC) estimated at around £5 m.

### Limitations

All the care homes involved in ENRICH had above-average assessments for the quality of care by the regulator. The network was able to recruit a range of care homes across a wide geographical spread but could not claim that they were representative. Nevertheless, ENRICH provided a wider base for recruitment to studies than has previously been the case. If, as was suggested by one participant, involvement in research is seen as an indicator for good care and a willingness to be open to scrutiny, it is possible that over time the expectations of the regulator, residents and their relatives will normalise participation in the network.

A key challenge to the success and sustainability of the network is the availability of studies to maintain care home participation and membership of the network, as well as those that they recognise as relevant to their work. It is a limitation of the evaluation that its scope and resources did not enable us to evaluate the longer term impact of ENRICH on recruitment to studies.

Delays in setting up the care home networks across all four local research networks in the regions, and the protracted research governance process seeking permissions in order to conduct interviews with care home managers, meant that it was not possible to set up focus groups with residents and their families, as originally planned. This illustrated one of the barriers to recruiting care homes identified by researchers in phase 1.

## Conclusions

This review of recent research to identify those studies that include care home residents revealed a small but increasing number of studies. Fostering and sustaining relationships with care homes appears to be the most efficient and effective way of recruiting them to the networks. The evaluation showed that it was possible to set up and run a number of research enabled care home networks using a variety of recruitment models, in a relatively short period of time. Based on these findings, recruitment target rates could work on the assumption that three months to recruit 10 care homes that are not part of a larger organisation is an achievable goal, if there is a prior history of association. Where this is not the case then it should be assumed that recruitment will be longer and will reflect the experiences of research teams recruiting for specific studies. However, once a care home is recruited to a network there is evidence to suggest that their involvement can be (and should be) retained.

Future research and development of ENRICH needs to consolidate and develop strategies that encourage reciprocity and relationship building with care homes. For on-going success, funders and researchers need to factor in enough time to recruit care homes and their residents and to provide training for researchers who do not have experience of care home research. They also need to include care home staff and owners in the research design and dissemination process to ensure they have greater involvement in setting research priorities and contributing to improved quality of care for residents.

## Abbreviations

CQC: Care Quality Commission; DeNDRoN: Dementias and Neurodegenerative Diseases Research Network; ENRICH: Enabling Research in Care Homes; ESRC: Economic and Social Research Council; MAGDR: Ministerial Advisory Group for Dementia Research; NIHR: National Institute for Health Research.

## Competing interests

All authors declare: no support from any organisation for the submitted work; no financial relationships with any organisations that might have an interest in the submitted work in the previous three years; no other relationships or activities that could appear to have influenced the submitted work.

## Authors’ contributions

CG, SLD, SI, JM, designed the protocol, SLD conducted online searches and interviews and screened studies for inclusion and extracted data for the phase one review. SLD recorded and evaluated the piloting of the ENRICH care home networks for phase two. CG and SLD wrote the paper. All authors interpreted the data and critically reviewed the paper.

## Pre-publication history

The pre-publication history for this paper can be accessed here:

http://www.biomedcentral.com/1471-2288/14/47/prepub

## Supplementary Material

Additional file 1: Table S1 List of care home research studies identified between March and July 2012. Click here for file
