# Supplementary material for: Enabling research in care homes: an evaluation of a national network of research ready care homes
Source: BMC Med Res Methodol. 2014 Apr 5;14:47. doi: 10.1186/1471-2288-14-47 (PMC4021218; doi:10.1186/1471-2288-14-47)
Supplement: Additional file 1: Table S1 — List of care home research studies identified between March and July 2012. [file 1471-2288-14-47-S1.docx]

**Additional file 1: Table S1 List of care home research studies identified between March and July 2012**

| **STUDY TITLE** | **Summary** | **Condition** | **Method – according to portfolio** | **STATUS** | **Global**  **Sample size** | | **Recruitment source** | **Other services involved** | **SOURCE** |
| --- | --- | --- | --- | --- | --- | --- | --- | --- | --- |
| 1. **Challenge Res Care** | **Examines the impact of training care home staff in behaviour management for dementia symptoms** | **Dementia** | **Interventional**  **- Web based training for care home staff** | **Open**  **30/04/13** | **1200**  **63 care homes**  **5-6 months to recruit** | | **Care homes only** | **No information** | **UKCRN portfolio** |
| 1. **Survey on long term care - RTPC** | **Development of best practice for transition from home care to care home for people with dementia** | **Dementia** | **Observational**  **- Survey**  **- Focus groups** | **Closed 31/03/12** | **2320**  **34 care homes in one study site** | | **Care home residents and older people at home** | **Community Mental Health Teams (CMHT)** | **UKCRN portfolio** |
| 1. **Wheld** | **Person centred intervention to improve mental health/reduce antipsychotics in care homes** | **Dementia** | **Intervention**  **- Person centred training for care home staff** | **Open**  **31/10/12** | **300**  **16 care homes in pilot study** | | **Care homes only** | **Psychologists, General Practitioners (GPs), pharmacists, Old age psychiatrists** | **UKCRN portfolio** |
| 1. **Nurses decisions to use anticipatory prescriptions** | **Role of community nurses in end of life medication decisions – includes nursing home nurses from up to 779 nursing homes** | **Cancer – end of life care** | **Observational**  **- Survey**  **- Interviews** | **Open**  **31/05/12** | **120**  **No residents included –**  **335+ nurses** | | **Nursing homes and District Nurse teams** | **Specialist nurses, GPs , pharmacists** | **UKCRN portfolio** |
| 1. **Shield – MCST implementation in practice** | **Psychosocial interventions in dementia including Cognitive Stimulation therapy (CST) staff training. Includes residents from care homes** | **Dementia** | **Interventional**  **CST training to staff so they can deliver it to residents with dementia** | **Open**  **28/09/12** | **340** | | **Awaiting information** | **No information** | **UKCRN portfolio** |
| 1. **Brain donation** | **Over 75s cohort study - brain donation for neuropathology** | **Dementia** | **Observational** | **Open**  **31/12/17** | **241 5/7 remaining are care home residents** | | **GP permission to contact possible participants** | **No information** | **UKCRN portfolio** |
| 1. **Cataract and cognition** | **Prospective longitudinal study of relationship between cataract and cognitive decline** | **Dementia** | **Observational**  **Clinical assessment and follow up** | **Open**  **01/08/12** | **120 1/85 recruited so far is a care home resident** | | **Secondary care**  **Via eye clinic** | **No information** | **UKCRN portfolio** |
| 1. **Evidem -E** | **Exercise as a therapy for BPSD**  **Walking intervention for older people with dementia and their carers** | **Dementia** | **Interventional**  **Randomised single blind controlled trial** | **Open**  **30/06/12** | **292 includes 15 residents from 10 care homes** | | **Dementia register /Psychiatric consultants** | **Approximately 15 care home staff** | **UKCRN portfolio** |
| 1. **The Hair and Care project** | **Personal identity and dementia care: improving care practice by exploring personal grooming in everyday lives of people with dementia Letters of invitation sent out by the home, family members also approached during visits** | **Dementia** | **Observational**  **Ethnographic**  **- interviews**  **-observation** | **Closed 30/03/12** | **20 – 10 people with dementia from care homes, and 8 care home staff** | | **Care homes and older people in their own homes 3 care homes 2 dual registered, 1 elderly mentally infirm, 1 withdrew but was replaced** | **No information** | **UKCRN portfolio** |
| 1. **OTCH** | **RCT occupational therapy intervention for residents with stroke in UK care homes** | **Parkinson’s Disease** | **Interventional**  **- Cluster randomised trial** | **Closed 28/02/12** | **1020** | | **572 residents recruited across 22 primary health care organisations from at least 22 care homes by 14 different researchers.** | **No information** | **UKCRN portfolio** |
| 1. **Main AD** | **RCT Memantine for the long term management of neuropsychiatric symptoms in AD** | **Dementia** | **Interventional**  **Multi centre randomised double blind clinical placebo controlled trial** | **10/06/11** | **300 240 care homes contacted. In one region 47 residents recruited across 27 care homes, ranging from 1-5 residents per care home** | | **Community Mental Health Teams** | **General Practitioners** | **UKCRN portfolio** |
| 1. **Main Ad sub-study** | **Genetic associations of behavioural and psychiatric systems of dementia** | **Dementia** | **Observational** | **31/12/12** | **90** | | **No information** | **No information** | **UKCRN portfolio** |
| 1. **MAGD Genes study** | **Sub study of MAGD - RCT of Memantine for agitation in AD - involved taking saliva DNA samples** | **Dementia** | **Observational** | **31/12/11** | **100 34 residents** | | **Database of patients who took part in MAGD clinical trial** | **No Information** | **UKCRN portfolio/**  **Email request** |
| 1. **Pitstop -** | **RCT of a complex intervention to prevent delirium in care homes for older people – aim to recruit all residents** | **(Psychosis )**  **All diseases** | **Interventional**  **Cluster randomised controlled pilot trial** | **In set up** | **300**  **12 care (+2 ) homes -30 contacted with nursing** | | **Care homes only – identified through CQC** | **CMHT** | **UKCRN portfolio** |
| 1. **Evidem eol** | **Changing practice in dementia care for end of life in care homes** | **Dementia** | **Interventional**  **- Participatory research approach for intervention** | **Closed 30/09/11** | **140 – 133 residents from 3 care homes** | | **Care homes only. All providing residential care** | **District Nurses, paramedics, Social care, GPs** | **UKCRN portfolio** |
| 1. **Care Med** | **Multi-professional medication reviews in care homes –RCT of cost effectiveness – 826 study participants** | **All diseases** | **Interventional**  **- Randomised controlled trial** | **Closed 12/12/11** | **30 – 2 nursing, 5 dual, 23 residential** | | **Care homes only but GPs that went into them had to agree to take part first** | **GPs, pharmacists** | **UKCRN portfolio** |
| 1. **Approach** | **A study to develop integrated working between primary health care services and care homes** | **All diseases** | **Observational** | **Closed**  **31/10/11** | **126**  **39 residents, from 6 care homes** | | **Care homes only 3 dual registered, 3 residential** | **District Nurses, specialist nurses, Community GPs, Therapists, Social Workers, Opticians** | **Phase 2 not on UKCRN portfolio** |
| 1. **Evidem - MCA** | **Changing practice in dementia care in the community. Developing practice and building evidence on the use of the MCA - 2005** | **Dementia** | **Observational** | **Closed**  **01/04/11** | **170 – 5 care homes nursing and residential** | | **Care homes only** | **33 care home staff recruited at time 1** | **UKCRN portfolio** |
| 1. **Fire – Facilitating implementation in Research Evidence** | **What helps to promote continence in older people in nursing homes and community hospitals? International study 4 countries including England – publically funded long term nursing care settings All residents over 60 with dementia and documented urinary incontinence** | **Continence** | **No information** | **4 year study** | **Staff consented as internal facilitators how many**  **No information on the number of residents in total** | | **Nursing homes** | **No information** | **Email request** |
| 1. **Improving end of life care for people with advanced dementia** | **Aims to understand the symptoms, health and social care needs of PWD and their informal carers and develop a complex intervention to improve care** | **End of life care** | **No information** | **Open**  **36 months** | | **12 care homes** | **No information** | **No information** | **Email request** |
| 1. **Preventing avoidable hospital admissions from care homes** | **pilot study to help develop a programme grant to design ways to reduce unnecessary hospital admissions in older people with frailty or dementia, living in care homes** | **Dementia** | **No information**  **Qualitative interviews and questionnaire** | **In set-up** | **40 (as an estimate)** | | **Recruiting 10 care homes – 9 nursing, 1 residential, 1 dual registered – 2 homes dropped out** | **No information** | **DENDRONportfolio** |
| 1. **The prevalence of visual impairment in dementia (PrOVIDe)** | **What is the prevalence of a range of vision problems in people with dementia aged 60-89 years and to what extent are these conditions undetected or inappropriately managed** | **Dementia** | **Observational**  **Eye tests** | **In set-up** | **770 – 385 living in own home and 385 living in care homes** | | **Memory clinics, in-patients, care homes** | **Possibly primary care** | **Dendron portfolio** |
| 1. **Care home residents’ experiences of moving care home** | **Care home residents’ experiences of the transitional phase of a move due to care home closure in the South West region of the UK.** | **No information** | **No information** | **Closed**  **31/01/11** | **No information** | | **No information** | **No information** | **Social care research register** |
| 1. **Manchester food in residential care project.** | **To assess how far residents in residential care homes demonstrate well-being during meal times.** | **All diseases** | **Observational** | **Closed**  **31/10/11** | **4 residential**  **care homes – 68 residents in total** | | **Info from conference presentation Care home only** | **Contract support services – catering** | **Social care research register** |
| 1. **Peach** | **Promoting excellence in all care homes exploring knowledge, practices and training needs of the care home workforce.** | **All diseases** | **Observational**  **Survey, focus groups and observation of communal practices in care homes** | **Closed**  **31/03/11** | **Survey sent to 1000 care home managers and 250 care workers** | | **No information** | **No information** | **Social care research register** |
| 1. **Understanding and implementation of the MCA** | **Generate an audit tool that focuses on integration, performance and consistency of the MCA with regard to day to day decisions. Involves visits to residential care homes in Essex.** | **No information** | **No information** | **Closed**  **30/02/11** | **No information given** | | **No information** | **No information** | **Social care research register** |
| 1. **Care home pathways and repeat hospital admissions in Essex** | **Part of larger study by ECC in collaboration with the Institute of Public Care to help inform strategies for prevention of hospitalisation of care home residents.** | **No information** | **No information** | **Closed**  **31/08/11** | **No information given** | | **No information** | **No information** | **Social care research register** |
| 1. **How reviewers implement review procedures and the impact it has on how residents experience reviews** | **Observation of 6 reviews in local authority homes and interviews with 6 older people who are the subject of the review** | **No information** | **No information** | **Open**  **31/07/12** | **6 care homes , 6 residents** | | **No information** | **No information** | **Social care research register** |
| 1. **(DCM EPIC Trial) Evaluating the effectiveness and cost effectiveness of Dementia Care Mapping (DMC) to enable person-centred care training (PCCT) for people with Dementia and Staff: A UK Cluster RCT in Care Homes** | **A full RCT of DMC as a care home intervention to support the implementation of PCCT)** | **Dementia** | **Randomised Controlled Trial of DMC** | **In set-up** | **750 residents**  **in 50 care homes** | | **Care homes only** | **No information** | **DeNDRoN portfolio** |
| 1. **Falls in Care Homes (FICH)** | **Feasibility study to inform a mains study of a falls prevention and training intervention:**  **Guide to Action for Care Homes (2) (GtACH)** | **Falls** | **Wearing accelerometer on leg and risk assessment** | **Open – May 2012** | **68** | | **Care homes only** | **No information** | **CSP under Age and Ageing** |
| 1. **Specialist healthcare Support for Older Residents in Care Homes – models and costs** | **To examine, in-depth, the organisation and operation of different models of service provision, and to estimate service costs based on known national unit costs** | **All diseases** | **Interviews and survey** | **Open – May 2012** | **15 services** | | **Care Homes** | **Primary Care Organisations** | **CSP under Age and Ageing** |
| 1. **Culture Change Studio Engagement Programme: Care home staff training** | **Latter to the Moon Culture Change Studio Engagement Programme – creative staff training to improve staff-resident interaction.** | **No information** | **Interventional**  **Focus groups, surveys** | **In set-up** | **172 – 100 staff, 32 residents and 40 relatives** | | **Care homes** |  | **CSP – under Age and Ageing** |
| 1. **PROF-COG Prevention of falls in older people with cognitive impairment older adults living in residential care homes** | **A pilot multi-factorial intervention to prevent falls in older people living in care homes tailored towards risk factors related to cognitive impairment** | **Falls** | **Interventional** | **In set-up** | **Information not available** | | **Information not available** | **Information not available** | **CSP under Age and Ageing** |

**Key: Details of care home involvement are highlighted in yellow**
